# A systematic review of the epidemiology of pediatric autoimmune encephalitis: disease burden and clinical decision-making

**DOI:** 10.3389/fneur.2024.1408606

**Published:** 2024-07-02

**Authors:** Jonathan D. Santoro, Panayotes Demakakos, Shiying He, Swati Kumar, Molly Murton, Frank Tennigkeit, Cheryl Hemingway

**Affiliations:** ^1^Department of Neurology, Keck School of Medicine of the University of Southern California, Los Angeles, CA, United States; ^2^UCB Pharma, Slough, United Kingdom; ^3^Costello Medical Singapore Ltd., Singapore, Singapore; ^4^Costello Medical Consulting Ltd., Cambridge, United Kingdom; ^5^UCB Biosciences, Monheim, Germany; ^6^Department of Neurology, Great Ormond Street Hospital for Children, London, United Kingdom; ^7^UCL Queen Square Institute of Neurology, London, United Kingdom

**Keywords:** autoimmune encephalitis, AIE, pediatric, epidemiology, systematic review

## Abstract

**Background:**

Autoimmune encephalitis (AIE) comprises a group of rare, immune system-mediated conditions. Clinical manifestations among children are not well-characterized, and there are challenges in testing and diagnosis. This can result in treatment delays, which has been found to correlate with poorer long-term outcomes. This challenge is exacerbated by the scarcity of epidemiological reporting of AIE. The objective of this systematic literature review (SLR) was to identify studies reporting epidemiological data on AIE in children.

**Methods:**

MEDLINE, Embase, the Cochrane Library, and the University of York Centre for Reviews and Dissemination (CRD) were searched in May 2023 for studies reporting on the epidemiology of AIE in children. These were supplemented with additional searches of conference proceedings, gray literature, and the reference lists of identified SLRs. Quality of studies was assessed using a modified version of the Joanna Briggs Institute (JBI) Checklist for Prevalence Studies.

**Results:**

Forty-three publications reporting on 41 unique studies were included. Nine studies reported incidence estimates of different subtypes of AIE, with only one reporting the incidence of overall AIE in children ≤ 18 years, estimated at 1.54 per million children per year in the Netherlands. Three studies reported the incidence of pediatric N-methyl-D-aspartate receptor (NMDAR)-AIE [in United Kingdom (UK), Hong Kong, and Denmark]. The other studies reported incidence data for selected populations.

**Conclusion:**

This SLR highlights a paucity of epidemiology data for AIE in children, which is likely reflective of difficulties in testing and diagnosis. There is a clear need for further research and awareness of these challenges in clinical practice to avoid treatment delays and improve patient outcomes. A deeper understanding of the epidemiology of AIE will help determine the worldwide burden of disease and inform research, health policies and clinical decision-making.

## 1 Introduction

Autoimmune encephalitis (AIE) comprises a group of rare, immune-mediated conditions where an individual’s immune system produces autoantibodies that target cells of the central nervous system (CNS) resulting in neuronal dysfunction, neuroinflammation, and cell-death (1). Multiple autoantibodies have been identified in individuals with AIE over the last two decades. The most prevalent are the anti-N-methyl-D-aspartate receptor (NMDAR), anti-myelin oligodendrocytic glycoprotein (MOG), anti-leucine-rich glioma inactivated 1 (LGI1), anti-contactin-associated protein 2 (CASPR2), anti-α-amino-3-hydroxy-5-methyl-4-isoxazolepropionic acid receptor (AMPAR), anti-gamma-aminobutyric acid A receptor (GABA_A_R), anti-metabotropic glutamate receptor (mGluR) and anti-glycine receptor (GlyR) autoantibodies, and some of them are often related with neoplasms (paraneoplastic autoantibodies) while others are not (2–4). The clinical manifestations of AIE are heterogenous and are temporally dependent on the location of the antigenic target in the CNS. Broadly, symptoms can include abnormal movements, behavioral changes, catatonia, cognitive dysfunction, confusion, memory loss, psychosis, and seizures (5), all of which could also be indicative of different autoimmune, neurologic or psychiatric conditions (1, 3). Aside from NMDAR-AIE, which is relatively well-characterized, similarities in imaging and laboratory findings make diagnosis and subtype determination of AIE challenging (3, 5). In addition to antibody-positive AIE, there is growing recognition of antibody-negative AIE, which includes cases of AIE that occur without any identifiable pathogenic antibody and present with similar clinical presentation as antibody-positive AIE (6).

Compared with adults, children with AIE may have different symptoms, paraclinical findings, comorbidities, treatment responses, and prognosis (4). The symptoms of AIE are also poorly characterized in children although important clinical differentiators have emerged. Children with AIE more frequently present with seizures, movement disorders, encephalopathy and multifocal neuropsychiatric symptoms, rather than insidious neurologic and psychiatric phenotypes (4, 5). Moreover, unlike adults, who often experience more subtle and fluctuant disease onset, children with AIE more frequently present as previously healthy with acute onset of neuropsychiatric symptoms (4, 5, 7).

Studies reporting the incidence and prevalence of AIE in children are scarce. This could in part be due to the challenges associated with the diagnosis of AIE in children, which relies on a combination of clinical history consistent with pediatric AIE and paraclinical and antibody testing (4). In antibody testing, both false-positive and false-negative results are possible, highlighting the importance of not relying solely on laboratory test results (8). As such, clinicians must synthesize a greater amount of data resulting in delays in diagnosis and treatment. Critically, these delays are associated with poorer long-term functional and cognitive outcomes, including worse verbal and visuospatial episodic memory scores (9–11). Early identification and diagnosis of AIE to avoid delays in treatment would therefore increase the likelihood of better longer-term outcomes.

A better understanding of the epidemiology of pediatric AIE and AIE subtypes could provide an estimate of the burden and distribution of AIE. This in turn could inform research, health policy, clinical decision-making guidelines, and allocation of resources. It can also aid more accurate diagnosis, for example by avoiding misdiagnosing patients with diseases that mimic AIE that are more prevalent in the general population (12, 13). Therefore, this systematic literature review (SLR) was conducted to identify published studies reporting epidemiological data on AIE (of any subtype) in children.

## 2 Methods

This SLR was conducted based on a prespecified protocol, in accordance with stringent methodological principles of conduct for SLRs (14, 15).

### 2.1 Identification of evidence

In May 2023, electronic database searches were conducted in MEDLINE, Embase, the University of York Centre for Reviews and Dissemination’s (CRD) Database of Abstracts of Reviews of Effects, the Cochrane Database of Systematic Reviews and the Cochrane Central Register of Controlled Trials. Manual searches of conference proceedings from the last 5 years (2019 to 2023) of six conferences (Encephalitis Conference; European Committee for Treatment and Research in Multiple Sclerosis; American Committee for Treatment and Research in Multiple Sclerosis; Child Neurology Society; Congress of the American Academy of Neurology; and the Congress of the European Academy of Neurology) were also performed. In addition, gray literature searches of regulatory websites, database aggregators and bibliographies of published SLRs were carried out.

Full details of all literature searches, including search strategies, are presented in Supplementary material. Articles were included in the SLRs if they met pre-specified eligibility criteria based on the Population, Intervention, Comparator, and Outcomes (PICO) framework (Table 1). Only studies that had an observational design and reported epidemiological outcomes including incidence, prevalence, population sizes, and geographic and temporal trends for children with AIE were included. Titles, abstracts, and relevant full texts were screened against the eligibility criteria by two independent reviewers. Any discrepancies were then discussed and resolved, and arbitrated by a third independent reviewer, if necessary. Screening of the supplementary sources was conducted by a single reviewer with a second reviewer providing input in cases of uncertainty and confirming all records for inclusion.

**Table 1:** Eligibility criteria for the SLR.

Category	Inclusion criteria	Exclusion criteria
Patient population	Children aged ≤ 18 years old with AIE of any subtype, including overall AIE	Population does not include children aged ≤18 years old with AIE of any subtype Mixed population of children and adults, unless outcomes are reported for children separately
Intervention/comparator	Any or none	N/A
Outcomes	Studies specifically designed to investigate one or more of: Incidence Prevalence (including point prevalence, partial prevalence and complete prevalence) Population size Geographic and temporal trends	Studies not reporting on any relevant outcomes
Study design	Observational studies (including prospective and retrospective long-term follow-up studies) SLRs and (N)MAs were included at the title/abstract review stage and hand-searched for additional relevant articles. They were excluded at the full-text review stage unless they themselves reported relevant primary data	Any other study design, including: RCTs Interventional non-RCTs Economic evaluations Non-systematic or narrative reviews Editorials, opinion pieces, notes or comments Case reports/case studies Trial protocols
Language	Abstracts or full texts in English	Non-English abstracts or full texts
Other considerations	Peer-reviewed journal articles Congress abstracts published in or since 2019 Studies in humans Any country, but calculated epidemiology focused on US and EU5 (France, Germany, Spain, Italy, UK), with extrapolations from other countries if necessary	Non peer reviewed studies Congress abstracts published prior to 2019 Studies not in humans

AIE, autoimmune encephalitis; EU5, European Union 5; N/A, not applicable; (N)MA, (network) meta-analysis; RCT, randomized controlled trial; SLR, systematic literature review; UK, United Kingdom; US, United States.

### 2.2 Calculation of incidence estimates using reported proportion and incidence data

In the case where studies reported proportion data (e.g., percentage of NMDAR-AIE among general AIE) rather than direct incidence rates, calculations were performed to generate incidence estimates. This was only possible if the denominator (i.e., the included study population, among which ‘X’ proportion had an AIE subtype) matched a population for which incidence data were available. The additional incidence estimates were calculated using the following approach, with further details described in Supplementary material:


$$\begin{array}{l} \mathrm{Reported}\;\mathrm{proportion}\;\mathrm{data}\;for\;a\;\mathrm{particular}\;\mathrm{subtype}\times \mathrm{reported}\;\\ \mathrm{overall}\;\mathrm{incidence}\;\mathrm{data}=\mathrm{incidence}\;\mathrm{estimate}\;for\;\mathrm{said}\;\mathrm{subtype}\text{.} \end{array}$$

Calculated incidence values are presented as lower and upper estimates, where possible, i.e., if more than one study reported the same proportion of a specific AIE subtype, both were used in the calculations to provide a range. Additionally, to facilitate comparisons across studies, all incidence estimates (both direct and calculated) were converted to a common unit, cases per million children per year. Full details of the incidence calculations are available in Supplementary material.

### 2.3 Data extraction and synthesis

Key information from each included study, including study characteristics, characteristics of the included patient population and epidemiological outcomes, was extracted into a pre-specified data extraction grid by a single individual. A second individual independently verified the extracted information. The quality of all included studies was assessed using a modified version of the JBI Checklist for Prevalence Studies (16). Quality assessments were completed by one individual and verified by a second independent reviewer.

## 3 Results

**Figure 1:**
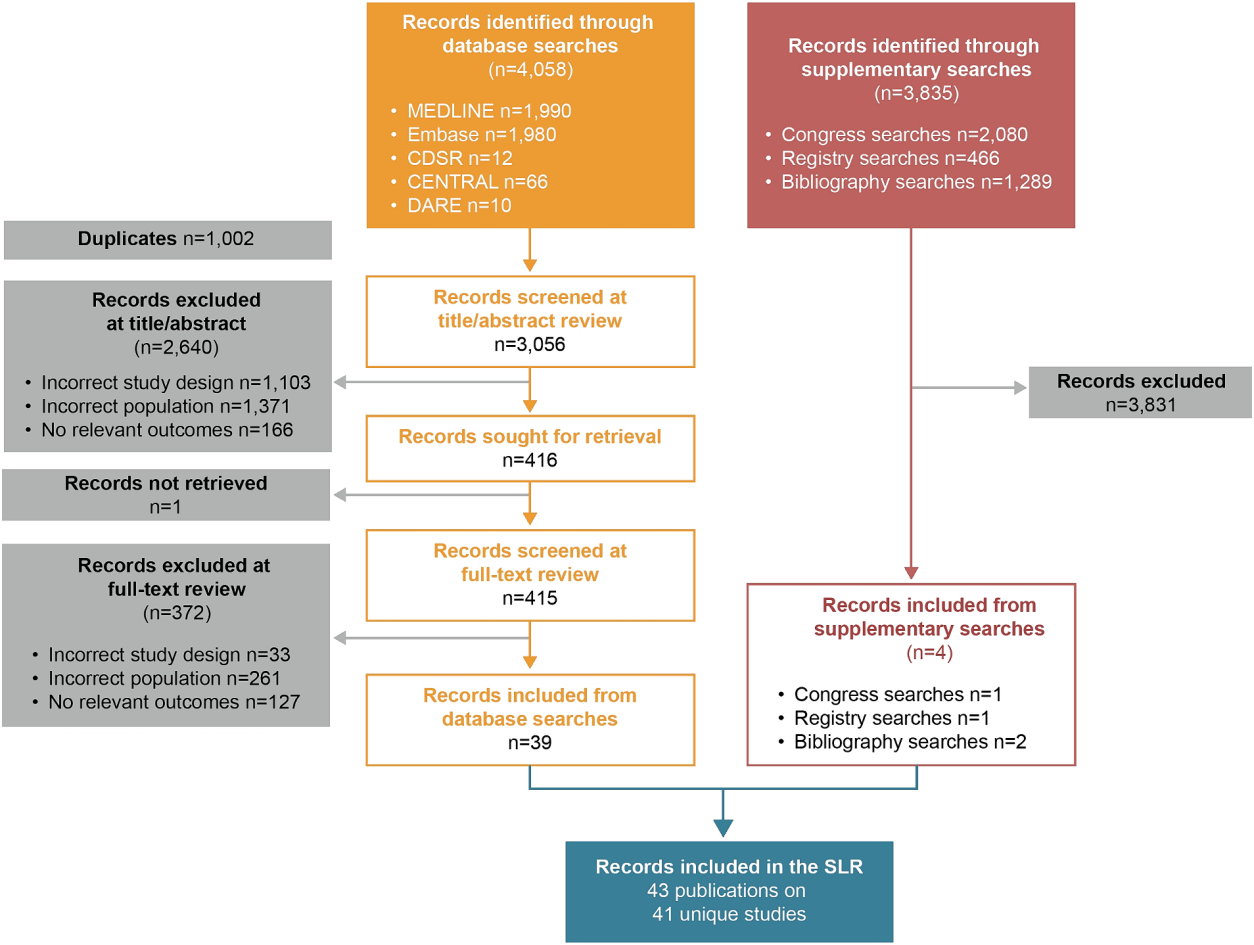
PRISMA flow diagram.

A Preferred Reporting Items for Systematic Reviews and Meta-Analyses (PRISMA) diagram displaying the flow of records through each stage of the review process is presented in Figure 1. A total of 4,058 records were retrieved by the electronic database searches. After exclusion of duplicate studies, 3,056 titles/abstracts (75.3%) were reviewed against the pre-specified eligibility criteria. Following this, 415 full-text articles (10.2%) were reviewed in full. Of these, 39 (1.0%) ultimately fulfilled the eligibility criteria for inclusion in the SLR. Supplementary searches yielded four additional records that fulfilled the eligibility criteria. In total, 43 publications reporting on 41 unique studies were included in the SLR.

Ten studies reported data on age of onset and/or presenting symptoms specific to the pediatric population with AIE (17–26). Age of onset of AIE ranged from 8 to 16 years for AIE overall, whereas age of onset for NMDAR-AIE ranged from 3.5–17 years in the identified studies. Age of onset was not reported for other AIE subtypes. The most common presenting symptoms, irrespective of AIE subtype, were seizures and behavioral change in combination with other neuropsychiatric features (17–25). These findings highlight the heterogenous presentation of AIE with little distinction among the different subtypes in the key presenting features.

### 3.1 Characteristics of studies reporting incidence

Nine studies that reported incidence estimates were identified (Table 2) (18, 20, 25, 27–32). Two were registry-based studies reporting data from Denmark and Malaysia (32), four were retrospective cohort studies from Asia Pacific and Canada (28–30), and three were prospective cohort studies from Europe (20, 25, 27). Sample sizes ranged from 16 to 375 children. The largest study was of the nationwide registry from Denmark, which included all children who underwent antibody testing between 2011 and 2017 (18). The other large study (*n* = 298) was a prospective United Kingdom (UK) cohort study that included children < 3 years presenting with new-onset epilepsy or complex febrile seizures between 2014 and 2017 (27).

**Table 2:** Summary of study characteristics for studies reporting incidence data identified in the SLR.

Study name	Study design	Setting	Country	Primary condition in population	No. of children included
**European studies**					
Boesen 2019 (18)	National registry/database	Multi-center	Denmark	AIE	Screened: 400
				<18 years	Enrolled: 375
Symonds 2020 (27)	Prospective cohort	Multi-center	Scotland	Children with new-onset epilepsy or complex febrile seizures	Screened: NR
				<3 years	Enrolled: 298
de Bruijn 2020 (20)	Prospective nationwide cohort	Multi-center	Netherlands	Included 3 different groups: (1) patients with definite AIE; (2) patients with ADEM, and (3) patients with suspected AIE	Screened: 113
				0–18 years	Enrolled: 103
Wright 2015 (25)	Prospective nationwide cohort	Multi-center	UK	NMDAR-AIE	Screened: NR
				1–17 years	Enrolled: 31
**North American studies**					
Parpia 2016^*^ (28)	Retrospective cohort	Multi-center	Canada	Encephalitis	Screened: NR
				Inclusion of children stated, specific age criteria NR	Enrolled: NR
**Asia Pacific studies**					
Fujii 2023 (29)	Retrospective cohort	Multi-center	Japan	GBS, FS, BBE	Screened: NR
				<15 years	Enrolled: 86
Jones 2017 (30)	Retrospective cohort	Multi-center	New Zealand	NMDAR-AIE	Screened: NR
				≤18 years	Enrolled: 16
Ho 2018 (31)	Retrospective cohort	Multi-center	Hong Kong	NMDAR-AIE	Screened: NR
				<18 years	Enrolled: 15
Keong Wong 2021^*^ (32)	National registry/database	Multi-center	Malaysia	NMDAR-AIE	Screened: NR
				Inclusion of children stated, specific age criteria NR	Enrolled: NR

ADEM, acute disseminated encephalomyelitis; AIE, autoimmune encephalitis; BBE, Bickerstaff brainstem encephalitis; FS, Fisher syndrome; GBS, Guillain-Barré Syndrome; NMDAR, N-methyl D-aspartate receptor; NR, not reported; UK. United Kingdom. ^*^Incidence reported directly for the pediatric population (<18 years) without reporting patient numbers or study reported the proportion of patients with AIE but did not report the number of patients enrolled in the study.

The type of incidence data reported varied across all nine studies. There were five studies that reported the incidence of AIE and/or AIE subtype out of the total population of children of a specific country or state (Table 3). Among these, two reported the incidence of overall AIE but only one considered children of any age. This was a Dutch nationwide study (20), which reported an incidence of 1.54 cases per million children per year for antibody positive AIE. Data were collected over a 4-year period (between 2015 and 2018) and reported incidence was based on the identification of 21 cases out of 34,089,992 Dutch children aged 0–18 years (20). The second study (28) also reported the incidence of immune-mediated AIE; however, this was restricted to incidence in children aged 1–4 years in Ontario, Canada. Reported incidence was 7.0 cases per million children per year (28). The remaining three studies (from the UK, Denmark, and Hong Kong) reported incidence of NMDAR-AIE within the pediatric population (18, 25, 31). Based on a pediatric population of 12 million in the UK, with 8 newly-diagnosed NMDAR cases from 2010 to 2011, an incidence estimate of 0.85 NMDAR-AIE cases per million children per year was reported by Wright 2015 (25). The studies from Denmark and Hong Kong had longer data collection periods (2011–2017 and 2009–2015, respectively) and reported higher incidence estimates, at 4.2 and 2.2 cases per million children per year, respectively (18, 31). The four studies that did not report incidence estimates out of the pediatric population included two that reported incidence estimates of NMDAR-AIE in specific ancestral or ethnic subpopulations (30, 32), and two that reported incidence estimates of other AIE subtypes, including post-infection AIE among children < 3 years with seizures, and Bickerstaff brainstem encephalitis (27, 29).

**Table 3:** Summary of key incidence data reported by identified studies.

Population incidence is reported in		Data collection period																	Cases per million children per year		Country	
		2002																2018
**Overall AIE**																					
Pediatric population	2015–2018																		1.54	Netherlands (20)	
Total Ontario population (1–4 years)	2002–2013																		7.0^*^	Canada (28)	
**NMDAR-AIE**																					
Pediatric population	2010–2011																		0.85	UK (25)	
Pediatric population	2009–2015																		2.2	Hong Kong (31)	
Pediatric population	2011–2017																		4.2^**^	Denmark (18)	
**Other AIE subtypes**																					
GAD65-AIE in pediatric population	2011–2017																		3.3^**^	Denmark (18)	
		Antibody negative AIE in pediatric population	2011–2017																			3.3**	Denmark (18)

AIE, autoimmune encephalitis; GAD65, Glutamic acid decarboxylase 65; NMDAR, N-methyl D-aspartate receptor; UK, United Kingdom. ^*^Incidence estimates were converted from per 100,000 children per year, ^**^Incidence estimates were converted from 100,000 person-years. The n/N estimates used to derive the incidence rates for these studies are as follows: 5/1,190,476 [NMDAR-AIE, (18)]; 4/1,212,121 [GAD65-AIE and antibody negative AIE, (18)]. Color shading indicate years when the study was conducted. The color shading depicts the year of data collection.

### 3.2 Calculated incidence estimates using reported proportion and incidence data

Ten studies that reported proportion data for AIE subtypes among definite AIE were used to calculate further incidence estimates (Table 4) (11, 20–24, 33–37). The calculated incidence estimates of AIE subtypes based on these proportion data are presented in Table 5. Based on studies reporting proportion data for NMDAR-AIE, annual incidence estimates (cases per million children per year) for pediatric NMDAR-AIE were calculated as: 0.4–1.4 in the Netherlands (19, 33), 1.4 in Hungary (34), 0.8 in the United States (US) (21, 22), 1.1–1.5 in China (23, 35), 0.3 in the Republic of Korea (24) and 0.3 in Australia (36). Using studies reporting proportion data for other AIE subtypes, incidence estimates were also calculated, including for LGI1-AIE in the Netherlands and China and Glutamic acid decarboxylase (GAD)-AIE in the UK and US (Table 5). In addition to AIE subtypes, incidence calculations were also performed for post-infection NMDAR-AIE in the Netherlands, Hong Kong and the Republic of Korea (Supplementary Tables 9, 11).

**Table 4:** Reported proportion data out of definite AIE (autoantibody positive) used in incidence calculations.

Definite AIE	Numerator	Denominator	No.^#^	Proportion (%)	Source
**Netherlands**					
Proportion	NMDAIR-AIE	All AIE subtypes identified	13	23.08	de Blauw 2020 (33)
	Hashimoto’s encephalitis			15.38	
	Rasmussen’s encephalitis			7.69	
	Other AIE^*^			7.69	
	NMDAR-AIE	All Dutch children with a confirmed diagnosis of AIE	21	90.48	de Bruijn 2020 (20)
	LGI1-AIE			4.76	
	AMPAR-AIE			4.76	
**United Kingdom**					
Proportion	GAD-AIE	Antibody positive AIE	21	9.52	Hacohen 2013 (11)
	GlyR AIE			4.76	
**Hungary**					
Proportion	NMDAR-AIE	Antibody positive AIE	8	75.00	Hayden 2021 (34)
	GABABR-AIE			12.50	
	LGI1-AIE + CASPR2 AIE			12.50	
**United States**					
Proportion	NMDAR-AIE	Autoimmune causes of encephalitis	60	51.67	Erickson 2020 (21)
	GAD-AIE			3.33	
	Hashimoto’s encephalitis			10.00	
	CV2/CRM5			1.67	
	NMDAR-AIE	Antibody positive AIE	19	52.63	Hariharan 2021 (22)
	GAD-AIE			21.05	

**China**					
Proportion	NMDAR-AIE	Patients with AIE admitted to Hunan Children’s Hospital over an 8-year period	103	94.17	Kang 2022 (23)
	LGI1-AIE			0.97	
	CASPR2-AIE			4.85	
	GABABR-AIE			1.94	
	NMDAR+CASPR2-AIE			0.97	
	CASPR2 + GABABR-AIE			0.97	
Proportion	NMDAR-AIE	Antibody positive AIE	70	70.00	Deng 2022 (35)
**Republic of Korea**					
Proportion	NMDAR-AIE	Pediatric AIE diagnosed using the criteria specified by Cellucci et al. (4)	110	21.82	Woo 2023 (24)
	MOG positive			15.45	
	Antibody negative AIE			24.54	
**Australia**					
Proportion	NMDAR-AIE	Autoantibody-associated AIE	56	17.86	Pillai 2015 (36)

AIE, autoimmune encephalitis; AMPAR, Alpha-amino-3-hydroxy-5-methyl-4-isoxazoleproprionic acid receptor, CASPR2, Contactin-associated protein-like 2; CV2, Cross veinless 2; CRM5, Collapsin response mediator protein 5; GABABR, Gamma-aminobutyric acid B receptor; GAD, Glutamic acid decarboxylase; GlyR, Glycine receptor; LGI1, Leucine-rich glioma inactivated 1; MOG, Myelin oligodendrocyte glycoprotein; NMDAR, N-methyl D-aspartate receptor. ^*^Patients for which autoimmune etiology had been demonstrated or was strongly suspected but the type of AIE was not clearly identified. ^#^Number is the number of patients in the given denominator.

**Table 5:** Summary of calculated incidence data (per million children per year).

Type of AIE	NMDAR	LGI1	AMPAR	Hashimoto’s encephalitis	Rasmussen’s encephalitis	GAD	GlyR	GABABR	CASPR2	LGI1 + CASPR2	NMDAR + CASPR2	CASPR2 + GABABR	MOG positive	Antibody negative	Dopamine D2R
**Estimates based on proportion in definite AIE cases (autoantibody positive)**															
Netherlands	0.4–1.4^*^	0.1	0.1	0.2	0.1	NC	NC	NC	NC	NC	NC	NC	NC	NC	NC
UK	NC	NC	NC	NC	NC	0.2	0.1	NC	NC	NC	NC	NC	NC	NC	NC
Hungary	1.4	NC	NC	NC	NC	NC	NC	0.2	NC	0.2	NC	NC	NC	NC	NC
US	0.8^**^	NC	NC	NC	NC	0.1–0.3^*^	NC	NC	NC	NC	NC	NC	NC	NC	NC
China	1.1–1.5^*^	0.01	NC	NC	NC	NC	NC	0.01	0.1	NC	0.01	0.01	NC	NC	NC
Republic of Korea	0.3	NC	NC	NC	NC	NC	NC	NC	NC	NC	NC	NC	0.2	0.4	NC
Australia	0.3	NC	NC	NC	NC	NC	NC	NC	NC	NC	NC	NC	NC	NC	0.1
Total	0.3–1.5	0.01–0.1	0.1	0.2	0.1	0.1–0.3	0.1	0.01–0.2	0.1	0.2	0.01	0.01	0.2	0.4	0.1

AIE, autoimmune encephalitis; AMPAR, Alpha-amino-3-hydroxy-5-m ethyl-4-isoxazoleproprionic acid receptor; CASPR2, Contactin-associated protein-like 2; D2R, Dopamine Receptor 2; GABABR, Gamma-aminobutyric acid B receptor; GAD, Glutamic acid decarboxylase; GlyR, Glycine receptor; LGI1, Leucine-rich glioma inactivated 1; MOG, Myelin oligodendrocyte glycoprotein; NC, not calculable; NMDAR, N-methyl D-aspartate receptor; UK, United Kingdom; US, United States. ^*^Lower and upper limit incidence estimates calculated using proportion data from different studies. ^**^Incidence estimates calculated using proportion data from different studies but resulted in the same value. The color shading depicts cells that have data reported.

### 3.3 Assessment of study quality of the included studies using the JBI critical appraisal checklist

The quality of the studies identified was moderate with mixed results across various domains, as determined by the JBI Checklist for Prevalence Studies. The results of the quality assessments for all studies are summarized in Supplementary material. The studies that specifically reported incidence data were judged to have conducted analysis with sufficient coverage, where all subgroups in the identified sample were judged to be represented (18, 20, 25, 27–32). All except one study (32) reported the criteria for defining AIE and/or valid methods for detecting antibodies in serum or cerebrospinal fluid (CSF). Three studies had missing data, which included one with only 50% response rate (29), one with incomplete reporting of incidence estimates by encephalitis cause (28), and one with unclear reporting of drop-outs (27). It was also found that all 11 studies reporting proportion data that were used to generate incidence estimates had conducted robust data analyses that included all patients in the sample (11, 19, 21–24, 33–36, 38). Nine out of the 11 studies reported valid methods and criteria used for the identification of AIE subtypes (11, 19, 21, 23, 33–36, 38) and all 11 reported adequate response rates. However, only three studies reported the study setting and patients adequately (11, 23, 38) and only five were considered to be representative of the target population (19, 23, 34, 35, 38).

## 4 Discussion

This SLR identified a distinct lack of published data on the epidemiology of pediatric-onset AIE. Only nine studies reporting incidence data were identified (18, 20, 25, 27–32), of which, only one reported incidence rates of definite AIE in a full pediatric population in children of all ages (20). Two studies reporting incidence estimates for NMDAR-AIE reported only on children in specific ancestral or ethnic subpopulations (30, 32). Only three studies reported results for specific AIE subtypes, including GAD-65 AIE and antibody-negative AIE (18); post-herpes simplex virus (HSV) infection AIE in children with seizures under 3 years (27), and Bickerstaff brainstem encephalitis (29). Reporting was sparse and disparate across geographies, with no incidence studies from the US, South America, Africa or Central Asia. Moreover, the quality of the evidence base was limited, with factors such as different periods of data collection and small sample size resulting in high between-study heterogeneity and thus limiting the confidence in the results. Although studies that reported proportion data for AIE were used to calculate further incidence estimates, these calculations were based on several assumptions, including the application of incidence rates from one country to another country, and thus only constitute crude estimates. As the symptoms of AIE have been found to be poorly-characterized in children, coupled with the reporting and severity bias associated with the studies included in this SLR, the calculated incidences are likely to be an underestimation of the true incidence of AIE (4).

One potential reason for the scarcity of data is that AIE in children may be under-recognized (39). Limited data on the condition’s epidemiology could be attributed to several reasons, including lack of clinical awareness, along with difficulties in AIE diagnosis and testing. In addition to its broad clinical phenotype, diagnosing AIE in children is difficult owing to the complexity of behavioral changes and the limited capacity of children to describe their symptoms (4). The diagnostic process is complex and requires comprehensive assessment, including clinical workup, neuroimaging, electroencephalogram (EEGs), neoplasia screening, and the collection of samples for testing, which can involve costly and invasive procedures which require sedation to obtain (3, 4). Moreover, antibody testing is generally associated with a number of limitations, and can result in both false-positive and false-negative results (8). Two studies identified in this SLR stated that they found no association between antibody positivity and presenting clinical phenotypes, with antibody negative groups presenting with similar clinical phenotypes to antibody positive groups (11, 27). This finding may be a result of delays between clinical presentation and antibody testing, or a reflection of the number of neural autoantibodies and their pathogenic mechanisms associated with AIE which have yet to be identified (4, 40). Given the growth of antibody testing panels in the past decade, it is critical to combine antibody tests with thorough clinical evaluation as the potential capture of novel autoantibodies may increase over time (4, 40).

Another important factor that may relate to the lack of published incidence data is the limited number of centers offering a dual antibody testing approach, which includes initial screening for antibodies using immunofluorescent assays followed by testing for specific antibodies using antibody titer assays and immunoblots, which provides an accurate diagnosis. However, this approach is currently only used by a limited number of international sites in the US and Europe and is not used for antibody testing in a number of other countries. Published information on exact costs for testing is difficult to obtain, but costs are known to differ by facility and region. Clinicians from other regions not offering testing (particularly Asia, South America, Africa) would be required to ship samples for long distances to these testing facilities that utilize more stringent methods for antibody detection or rely solely on the results of commercially available assays. This aligns with the studies identified in this SLR, with the majority using commercial testing kits rather than samples being tested at a specialized facility offering this dual testing. Concerns have been raised regarding false negative results in certain commercial assays and antibody kits, especially when CSF alone is used as a sample. This is more predominantly seen for LGI1, GABABR and AMPAR antibodies. This highlights the fact that any inferences made on the incidence of AIE from these studies might not be as accurate (41). A substantial number of healthcare institutions face barriers in accessing these testing facilities, which can result in patients going with unconfirmed or inaccurate AIE diagnoses, thereby contributing to the paucity of epidemiological data on antibody-mediated AIE.

Finally, there may be differences in the approach taken to treat AIE in children. The 2020 guidelines for differential diagnosis of AIE in children by Cellucci et al. (4) highlights the importance of starting therapy while awaiting the results of antibody testing among children suspected to have AIE. Expert consensus is consistent with this recommendation; in a 2018 report on the expert opinion of three AIE specialists from three different continents (US, UK, and India) regarding the challenges of AIE diagnosis and the role of antibody testing, the experts emphasized the importance of starting treatment for suspected AIE cases while awaiting the results of antibody testing, without being over reliant on test results (42). However, awareness of this urgency to initiate treatment may be lacking among less experienced or non-specialist clinicians. A worldwide survey of 1,333 neurologists from 94 countries asked questions on their approach to dealing with AIE cases (43). In response to clinical questions about a patient strongly suspected of AIE, the majority of clinicians responded that they would treat the patient empirically for presumed AIE, while 11% would wait for antibody test results. However, for ambiguous cases that had partial phenotypes, only 40% of respondents said that they would consider empiric immunotherapy, and 28% would only do so on confirmatory antibody test results. Unsurprisingly, these decisions were found to be associated with the number of AIE cases seen annually by the treating physician, where more experienced physicians chose to start treatment early. This finding highlights the rarity of AIE and how this impacts the ability of physicians to recognize and treat the condition appropriately. It also emphasizes the importance of epidemiological data in gaining a deeper understanding of the disease burden and how this affects clinical decision-making. Physician likelihood-to-treat discrepancies due to not seeing patients with AIE frequently are thus compounded by the limitations in access to autoantibody testing and by cognitive biases to not treat individuals who do not have known autoantibody mediated AIE. Multiple studies have found that delays in diagnosis, including waiting times for antibody test results, can result in unnecessary delays in treatment, which has been found to be associated with poorer long-term outcomes (9–11). To improve the odds of favorable outcomes for patients, there is a critical need for earlier diagnosis and aggressive treatment of AIE. Given the emphasis on early treatment initiation prior to antibody test confirmation, clinicians should prioritize prompt initiation of immunosuppressive therapy for AIE while waiting for antibody confirmation. Following the receipt of antibody testing results, the treatment strategy could be altered or refined to suit the patient’s needs (5). This approach should be utilized until further real-world data on the epidemiology of AIE becomes available to better inform treatment strategies. A deeper understanding of the epidemiology of AIE is also essential to inform research, health policies and clinical decision-making guidelines at a global scale.

One of the best examples of how epidemiological data can be instrumental in making decisions for better allocation of resources for prevention and treatment of diseases is the recent COVID-19 pandemic. Epidemiological studies published in the months following the outbreak of the virus highlighted that worse COVID-19-related outcomes were positively correlated with people aged ≥ 60 years old and those with underlying co-morbidities (44–46). This identified a vulnerable population to be prioritized for vaccination drives, implementation of stricter regulations for self-isolation and distancing. Epidemiological data were also considered when deciding which patients should be fully escalated to invasive mechanical ventilation or other types of organ support. Furthermore, research into the epidemiology of anti-NMDAR encephalitis following the condition’s discovery increased clinician awareness of the disease phenotype and aided in treatment decision making for patients presenting with symptoms including rapidly progressive psychiatric symptoms or cognitive impairment, seizures, abnormal movements, or coma of unknown cause (47).

### 4.1 Strengths and limitations

This SLR was conducted in accordance with stringent methodology, including an exhaustive literature search as well as independent dual review of studies against pre-specified eligibility criteria to minimize the risk of selection bias. However, there were some limitations to the methodology and evidence base. For instance, only studies with abstracts or full texts written in English were included, which may have resulted in the omission of relevant data published in other languages. Furthermore, only descriptive synthesis was performed in this SLR, and interpretations were based on ranges of reported outcomes data rather than relying on statistical adjustment or modeling, due to the scarcity and heterogeneity of evidence identified. While calculations were conducted to estimate more incidence data, they were based on several assumptions. For example, given that only one study from the Netherlands reported incidence estimates of definite AIE, this estimate was assumed to be generalizable across country settings (including countries in Asia) and subsequently used to derive incidence estimates of AIE subtypes of other countries. Moreover, due to the scarcity of data, it was not possible to make adjustments to reported data to address the likely issue of between-study heterogeneity (including variation in baseline characteristics, years of data collection, diagnostic criteria used and methods of antibody detection). Finally, as the incidence calculations were based on reported studies in the literature wherein reporting, selection, and severity bias were present, the incidence estimates generated are likely to reflect an underestimation of the real-world incidences of AIE. Nonetheless, this SLR has provided a much-needed overview of the published epidemiological data for AIE in children and revealed the need for high fidelity, nationwide and international registry studies, to enable across-country comparisons and improve the systematic data capture of children with AIE.

## 5 Conclusion

Overall, the findings of this SLR support that pediatric AIE is very rare. However, given the multiple biases present in the reported literature included in this SLR, the calculated incidence estimates presented here likely underestimate the true incidence of AIE in children. Further research at a national/international level, across a wider breadth of countries is critically necessary. With broader awareness and identification of AIE in clinical practice extracted from these types of studies, earlier diagnosis and treatment may lead to improved long-term outcomes in this unique patient population. Moreover, epidemiological data will play an instrumental role in informing research, health policies, clinical decision-making and also to better understand the worldwide burden of disease.

## Data availability statement

The original contributions presented in the study are included in the article/Supplementary material, further inquiries can be directed to the corresponding author.

## Author contributions

JS: Conceptualization, Formal analysis, Investigation, Methodology, Supervision, Visualization, Writing – review & editing, Writing – original draft. PD: Conceptualization, Investigation, Methodology, Supervision, Visualization, Writing – review & editing, Writing – original draft. SH: Conceptualization, Formal analysis, Investigation, Methodology, Visualization, Writing – original draft, Writing – review & editing, Project administration. SK: Conceptualization, Formal analysis, Investigation, Methodology, Visualization, Writing – original draft, Writing – review & editing, Project administration. MM: Conceptualization, Formal analysis, Investigation, Methodology, Supervision, Visualization, Writing – original draft, Writing – review & editing. FT: Conceptualization, Formal analysis, Investigation, Methodology, Supervision, Visualization, Writing – review & editing, Writing – original draft, Funding acquisition. CH: Conceptualization, Formal analysis, Investigation, Methodology, Supervision, Visualization, Writing – review & editing, Writing – original draft.
