# Supplementary material for: A systematic review of the epidemiology of pediatric autoimmune encephalitis: disease burden and clinical decision-making
Source: Front Neurol. 2024 Jul 2;15:1408606. doi: 10.3389/fneur.2024.1408606 (PMC11262030; doi:10.3389/fneur.2024.1408606)
Supplement: Supplementary file 1 [file Data_Sheet_1.docx]

*SUPPLEMENTARY MATERIALS*

Supplementary Table 1. Search terms for use in MEDLINE (searched via the Ovid SP platform).

| **Dates searched: 1946 to May 18^th^ 2023**  **Records retrieved: 1990** | | | |
| --- | --- | --- | --- |
| **Term group** | **#** | **Search terms** | **Results: 22^nd^ May 2023** |
| **Autoimmune encephalitis** |  | *Encephalitis/ or *Limbic Encephalitis/ or *Paraneoplastic Polyneuropathy/ or *Autoimmune Diseases of the Nervous System/ or Anti-N-Methyl-D-Aspartate Receptor Encephalitis/ | 19612 |
|  |  | (autoimmune encephalitis or auto-immune encephalitis or paraneoplastic encephalitis or limbic encephalitis or antibody encephalitis).ti,ab,kf. | 4392 |
|  |  | ((brain or cerebral) adj2 (inflamm$ or swell$)).ti,ab. | 7516 |
|  |  | ((autoantibod$ or antibod$ or autoimmune$ or auto-immun$) adj3 (encephalitis or epilep$ or seizure$ or convuls$)).ti,ab. | 3910 |
|  |  | or/1-4 | 29196 |
| **Epidemiological studies (Objective 1)** |  | *Epidemiologic studies/ or ep.fs. | 2095561 |
|  |  | (epidemiolog$ adj (study or studies)).ti,ab,kf. | 100052 |
|  |  | (epidemiolog$).ti. | 133555 |
|  |  | *incidence/ or *prevalence/ or (inciden$ or prevalen$).ti,ab,kf./freq=2 | 747248 |
|  |  | or/6–9 | 2524140 |
| **LGI1-AIE (Objective 2)** |  | (anti$ adj (LGI1 or "LGI 1" or leucine$)).ti,ab,kf. | 328 |
|  |  | (((LGI1 or "LGI 1" or leucine$) adj5 (encephalitis or epilepsy)) or Morvan syndrome).ti,ab,kf. | 534 |
|  |  | or/11-12 | 633 |
| **Limits** |  | exp Animals/ not exp Humans/ | 5122532 |
|  |  | (comment or editorial or historical article).pt. | 1806628 |
|  |  | or/14–15 | 6870509 |
| **Total** |  | 5 and (10 or 13) | 2230 |
|  |  | 17 not 16 | **1990** |

**Database**: Ovid MEDLINE(R) and Epub Ahead of Print, In-Process, In-Data-Review & Other Non-Indexed Citations and Daily 1974 to May 19th 2023. **Abbreviations:** AIE, autoimmune encephalitis; LGI1, Leucine-rich glioma inactivated 1.

Supplementary Table 2. Search terms for use in Embase (searched via the Ovid SP platform).

| **Dates searched: 1974 to May 19^th^ 2023**  **Records retrieved: 1980** | | | |
| --- | --- | --- | --- |
| **Term group** | **#** | **Search terms** | **Results: 22^nd^ May 2023** |
| **Autoimmune encephalitis** |  | *encephalitis/ or *limbic encephalitis/ or *paraneoplastic encephalitis/ or exp autoimmune encephalitis/ | 18738 |
|  |  | (autoimmune encephalitis or auto-immune encephalitis or paraneoplastic encephalitis or limbic encephalitis or antibody encephalitis).ti,ab,kf. | 7763 |
|  |  | ((brain or cerebral) adj2 (inflamm$ or swell$)).ti,ab. | 10488 |
|  |  | ((autoantibod$ or antibod$ or autoimmune$ or auto-immun$) adj3 (encephalitis or epilep$ or seizure$ or convuls$)).ti,ab. | 6918 |
|  |  | or/1-4 | 34602 |
| **Epidemiological studies (Objective 1)** |  | *Epidemiology/ or ep.fs. | 1203326 |
|  |  | (epidemiolog$ adj (study or studies)).ti,ab,kf. | 131867 |
|  |  | (epidemiolog$).ti. | 154454 |
|  |  | *incidence/ or *prevalence/ or (inciden$ or prevalen$).ti,ab,kf./freq=2 | 1129350 |
|  |  | or/6–9 | 2201201 |
| **LGI1-AIE (Objective 2)** |  | (anti$ adj (LGI1 or "LGI 1" or leucine$)).ti,ab,kf. | 533 |
|  |  | (((LGI1 or "LGI 1" or leucine$) adj5 (encephalitis or epilepsy)) or Morvan syndrome).ti,ab,kf. | 858 |
|  |  | or/11-12 | 1033 |
| **Limits** |  | ("conference abstract" or "conference review").pt. | 4781671 |
|  |  | limit 14 to yr="1974–2017" | 3038123 |
|  |  | exp animal/ not exp human/ | 5178640 |
|  |  | editorial.pt. | 775389 |
|  |  | editorial/ | 744495 |
|  |  | or/15–18 | 8741398 |
| **Total** |  | 5 and (10 or 13) | 2370 |
|  |  | 20 not 19 | 1980 |

**Database**: Embase 1974 to May 19^th^ 2023. **Abbreviations:** AIE, autoimmune encephalitis; LGI1, Leucine-rich glioma inactivated 1.

Supplementary Table 3. Search terms for use in CDSR (searched via the Ovid SP platform).

| **Dates searched: 2005 to May 16^th^ 2023**  **Records retrieved: 12** | | | |
| --- | --- | --- | --- |
| **Term group** | **#** | **Search terms** | **Results: 22^nd^ May 2023** |
| **Autoimmune encephalitis** |  | encephalitis.kw. or limbic encephalitis.kw. or paraneoplastic polyneuropathy.kw. or autoimmune diseases of the nervous system.kw. or anti-n-methyl-d-aspartate receptor encephalitis.kw. | 8 |
|  |  | (autoimmune encephalitis or auto-immune encephalitis or paraneoplastic encephalitis or limbic encephalitis or antibody encephalitis).ti,ab,kw. | 0 |
|  |  | ((brain or cerebral) adj2 (inflamm$ or swell$)).ti,ab. | 6 |
|  |  | ((autoantibod$ or antibod$ or autoimmune$ or auto-immun$) adj3 (encephalitis or epilep$ or seizure$ or convuls$)).ti,ab. | 0 |
| **Total** |  | or/1-4 | **12** |

**Database**: EBM Reviews - Cochrane Database of Systematic Reviews 2005 to May 16th 2023. **Abbreviations:** CDSR, Cochrane Database of Systematic Reviews.

Supplementary Table 4. Search terms for use in the CENTRAL (searched via the Ovid SP platform).

| **Dates searched: 22^nd^ May 2023**  **Records retrieved: 66** | | | |
| --- | --- | --- | --- |
| **Term group** | **#** | **Search terms** | **Results: 22^nd^ May 2023** |
| **Autoimmune encephalitis** |  | Encephalitis/ or Limbic Encephalitis/ or Paraneoplastic Polyneuropathy/ or Autoimmune Diseases of the Nervous System/ or Anti-N-Methyl-D-Aspartate Receptor Encephalitis/ | 229 |
|  |  | (autoimmune encephalitis or auto-immune encephalitis or paraneoplastic encephalitis or limbic encephalitis or antibody encephalitis).ti,ab,kw. | 55 |
|  |  | ((brain or cerebral) adj2 (inflamm$ or swell$)).ti,ab. | 285 |
|  |  | ((autoantibod$ or antibod$ or autoimmune$ or auto-immun$) adj3 (encephalitis or epilep$ or seizure$ or convuls$)).ti,ab. | 74 |
|  |  | or/1-4 | 557 |
| **Epidemiological studies (Objective 1)** |  | Epidemiologic studies/ or ep.fs. | 57069 |
|  |  | (epidemiolog$ adj (study or studies)).ti,ab,kw. | 4612 |
|  |  | (epidemiolog$).ti. | 1215 |
|  |  | incidence/ or prevalence/ or (inciden$ or prevalen$).ti,ab,kw./freq=2 | 96478 |
|  |  | or/6–9 | 137142 |
| **LGI1-AIE (Objective 2)** |  | (anti$ adj (LGI1 or "LGI 1" or leucine$)).ti,ab,kw. | 1 |
|  |  | (((LGI1 or "LGI 1" or leucine$) adj5 (encephalitis or epilepsy)) or Morvan syndrome).ti,ab,kw. | 7 |
|  |  | or/11-12 | 7 |
| **Limits** |  | Animals/ not Humans/ | 2672 |
|  |  | (comment or editorial or historical article).pt. | 5860 |

**Database:** EBM Reviews - Cochrane Central Register of Controlled Trials April 2023. **Abbreviations:** AIE, autoimmune encephalitis; LGI1, Leucine-rich glioma inactivated 1.

Supplementary Table 5. Search terms for use in DARE (searched via the Ovid SP platform)

| **Dates searched: 1^st^ Quarter 2016**  **Records retrieved: 10** | | | |
| --- | --- | --- | --- |
| **Term group** | **#** | **Search terms** | **Results: 22^nd^ May 2023** |
| **Autoimmune encephalitis** |  | encephalitis.kw. or limbic encephalitis.kw. or paraneoplastic polyneuropathy.kw. or autoimmune diseases of the nervous system.kw. or anti-n-methyl-d-aspartate receptor encephalitis.kw. | 9 |
|  |  | (autoimmune encephalitis or auto-immune encephalitis or paraneoplastic encephalitis or limbic encephalitis or antibody encephalitis).ti. | 0 |
|  |  | ((brain or cerebral) adj2 (inflamm$ or swell$)).ti. | 1 |
|  |  | ((autoantibod$ or antibod$ or autoimmune$ or auto-immun$) adj3 (encephalitis or epilep$ or seizure$ or convuls$)).ti. | 0 |
| **Total** |  | or/1-4 | **10** |

**Database:** EBM Reviews - Database of Abstracts of Reviews of Effects to 1st Quarter 2016.

Supplementary File 1. Detailed Overview Epidemiology Calculations

Extracted epidemiological outcomes including incidence of pediatric AIE or AIE subtypes in a general population were used to estimate the incidence of AIE and their subtypes across geographical regions. Incidence estimates were converted to a common unit: cases per million children per year, to facilitate comparisons across studies. For studies that reported estimates as cases per 100,000 children per year (based on regional or national pediatric population numbers), this was simply multiplied by 1,000,000 and divided by 100,000 to convert the estimate to per million children per year. For studies that reported estimates as cases per 100,000 person-years but did not report the population size used to derive their incidence estimates, these population sizes were first estimated using the following formula:([1](#_ENREF_1))

**(number of new cases * 100,000) / (population size * study duration) = cases per 100,000 person-years**

Rearranged to calculate population size: **population size = number of new cases * 100,000 / (study duration * cases per 100,000 person-years)**

The estimated population size was then used to calculate the incidence per million children per year by dividing the number of new cases reported by the calculated population size and then multiplying this by 1,000,000. Further to these calculations, which were simply based on estimates reported across the identified studies, overall population numbers for Denmark and Scotland were sourced from Statistics Denmark for 1^st^ January 2018 and Scotland’s Census for 2022, respectively, to derive additional incidence estimates and values were compared (**Supplementary Table 6**) ([2](#_ENREF_2), [3](#_ENREF_3)). Worked examples of how this approach was used in the calculations is described in **Supplementary File 2**.

Given the limited reporting of incidence data, proportion data were assessed for their appropriateness for use in calculations. All extracted epidemiological outcomes and proportion data were screened for populations with confirmed autoantibody positivity, either at study entry or via a confirmatory result after autoantibody testing during the study. The data could only be used if the denominator (i.e., the included study population, among which 'X' proportion had AIE) matched a population for which reported incidence data were available. Calculations were then performed using the following approach:

**Reported proportion data for a particular subtype * reported overall incidence data = incidence estimate for said subtype**

If more than one source reported the same type of incidence or same type of proportion data, multiple calculations were performed to estimate an upper and lower limit of the possible incidence.

A total of 39 studies were identified that reported on proportion data out of which 11 studies were used in the calculations. As incidence estimates from identified studies were only available for definite AIE (autoantibody positive) and NMDAR-AIE in the general population, only studies that reported proportion data with these specific populations as the denominator were considered for the epidemiology calculations. Calculated incidence values are presented as lower and upper estimates, where possible i.e., if more than one study reported the same proportion of a specific AIE subtype, both were used in the calculations to provide a range. Worked examples of the calculations performed is included in **Supplementary File 3**.

Supplementary File 2. Worked Example for Epidemiology Calculations (Studies Reporting Incidence Data)

Based on Supplementary Figure 2, the following information was reported in Boesen 2019: ([4](#_ENREF_4))

1. New incident cases of NMDAR-AIE: 5
2. Years of data collection: 6
3. Incidence in 100,000 person-years: 0.07

As the overall population size used to calculate this incidence rate is not reported, manual calculations were performed to convert the reported incidence rate to per million per year.

1. The following formula was used: (number of new cases * 100,000) / (population size * study duration in years) = cases per 100,000 person-years
2. The formula was then rearranged to calculate population first as this was missing from the study: population size = number of new cases * 100,000 / (study duration in years * cases per 100,000 person-years)
3. This formula was applied the Boesen study to derive the overall population (number): population size = 5 * 100,000 / (6 * 0.07) = 1,190,476.19
4. The incidence per million per year was then calculated: number of new cases / calculated population * 1,000,000 = 5 / 1,190,476.19 * 1,000,000 = 4.2

**Incidence Calculations using Population Estimates from Online Sources and Comparison with Estimates Calculated using the Formula**

The formula stated above was used to estimate incidence for two studies (Boesen 2019 and Symonds 2020) based in Denmark and Scotland, respectively.([4](#_ENREF_4), [5](#_ENREF_5))

Incidence estimates for these studies were also calculated using population estimates derived from online sources. These estimates for Denmark and Scotland were derived from Statistics Denmark and Scotland’s Census respectively as follows:([2](#_ENREF_2), [3](#_ENREF_3))

1. Population estimate (<18 years) for Denmark for 1^st^ January 2018 (study end-date was 27^th^ December 2017): 1,165,500
2. Population estimate (0–4 years) for Scotland for 2022: 247,100
3. The incidence per million per year was then calculated: number of new cases reported in the respective studies / population estimates derived from online sources * 1,000,000
4. New cases of NMDAR-AIE reported in Boesen 2019: 5
5. The incidence per million per year: number of new cases reported in the respective studies/ population estimates derived from online sources * 1,000,000 = 5 / 1,165,500 * 1,000,000 = 4.3
6. The same approach was used to calculate incidence of antibody negative AIE (Boesen 2019) and post-HIV infection AIE (Symonds 2020)

Supplementary Table 6. Comparison of incidence estimates calculated using the formula to determine population and population estimates directly derived from online sources.

| **Numerator (incidence data for)** | **Denominator (data out of)** | **Year range** | **Incidence estimates calculated using formula*** | **Incidence estimates calculated using population estimates derived*** | **Study Name** |
| --- | --- | --- | --- | --- | --- |
| NMDAR-AIE | Pediatric population in Denmark | 2011–2017 | 4.2 | 4.3 | Boesen 2019 ([4](#_ENREF_4)) |
| Antibody-negative AIE | Pediatric population in Denmark |  | 3.3 | 3.4 |  |
| GAD-65 AIE | Pediatric population in Denmark |  | 3.3 | 3.4 |  |
| Post-HSV infection AIE (<3 years) | Children with seizures | 2014–2017 | 5.7 | 4.0 | Symonds 2020 ([5](#_ENREF_5)) |

**Abbreviations**: AIE, autoimmune encephalitis; GAD, Glutamic acid decarboxylase; HSV, herpes simplex virus; NMDAR, N-methyl D-aspartate receptor. **Footnotes:** *Incidence reported as per million children per year.

Supplementary File 3. Worked Example for Epidemiology Calculations (Studies Reporting Proportion and Incidence Data)

De Bruijn et al. (2020) reported incidence for overall AIE, but additionally within the patient characteristics reported proportions of different subtypes, enabling a crude calculation of an estimate of the likely incidence rate for those subtypes ([6](#_ENREF_6)).

Calculations were then performed using the following approach:

1. Reported proportion data for a particular subtype multiplied by reported overall incidence data = incidence estimate for said subtype
2. Proportion (%) of NMDAR-AIE from de Bruijn 2020: NMDAR-AIE cases / Definite-AIE cases * 100 = 19 / 21 * 100 = 90.5
3. Reported Incidence Data for definite-AIE: 1.54 per million children per year
4. Incidence Estimate for NMDAR-AIE (using the above formula): 90.5 / 100 * 1.54 = 1.4

Calculations for NMDAR-AIE for de Bruijn 2020 were also estimated using the population estimates reported in the study (as seen in **Supplementary Table 11**) using the formula described previously, to compare the estimates derived using reported proportion data:

1. Number of new cases / reported population size * 1,000,000 = 19 / 3,408,992 = 5.6
2. This was then divided by 4 to consider the average population across 4 years = 5.6 / 4 = 1.3

Supplementary Table 7. Summary of other incidence data reported by identified studies.

| **Numerator (incidence data for)** | **Denominator (data out of)** | **Year range** | **Incidence per million children per year** | **Study name** | **Country** |
| --- | --- | --- | --- | --- | --- |
| **NMDAR-AIE (n=2)** | | | | | |
| NMDAR-AIE with Maori or Pacific ancestry | Children with Maori ancestry in New Zealand | 2008–2015 | 3.4 | Jones 2017  ([7](#_ENREF_7)) | New Zealand |
| NMDAR-AIE with Maori or Pacific ancestry | Children with Pacific ancestry in New Zealand |  | 10 |  |  |
| NMDAR-AIE with Maori or Pacific ancestry | Children without Maori or Pacific ancestry in New Zealand |  | 0.2 |  |  |
| NMDAR-AIE | Overall pediatric population who are Austronesians in Malaysia | 2015–2019 | 3.63 | Keong Wong 2021  ([8](#_ENREF_8)) | Malaysia |
| NMDAR-AIE | Overall pediatric population who are Chinese in Malaysia |  | 2.59 |  |  |
| **Other AIE subtypes (n=3)** | | | | | |
| Post-HSV infection AIE (<3 years) | Children <3 years with seizures in Scotland | 2014–2017 | 5.7** | Symonds 2020 ([5](#_ENREF_5)) | Scotland |
| GAD65-AIE | Pediatric population in Denmark | 2011–2017 | 3.3** | Boesen 2019 ([4](#_ENREF_4)) | Denmark |
| Antibody negative AIE | Pediatric population in Denmark |  | 3.3** |  |  |
| Bickerstaff brainstem encephalitis | Average childhood population in Japan | 2014–2016 | 0.071* | Fujii 2023 ([9](#_ENREF_9)) | Japan |

**Abbreviations:** AIE, autoimmune encephalitis; GAD65, Glutamic acid decarboxylase 65; HSV, herpes simplex virus; NMDAR, N-methyl D-aspartate receptor. **Footnotes**: ^*^Incidence estimates were converted from per 100,000 children per year, **Incidence estimates were converted from 100,000 person-years.

Supplementary Table 8: Summary of study characteristics for studies reporting proportion data used in the calculation of incidence estimates.

| **Study name** | **Study design** | **Single-center/ multi-center** | **Continent** | **Countries** | **Primary condition in population** | **No. of children included/total population** |
| --- | --- | --- | --- | --- | --- | --- |

| **de Blauw 2020 (**[**10**](#_ENREF_10)**)** | Retrospective cohort | Multi-center | Europe | Netherlands | Encephalitis | 121/121 (children <18 years)  Screened: 161; Enrolled: 121 |
| --- | --- | --- | --- | --- | --- | --- |
| **Hacohen 2013 (**[**11**](#_ENREF_11)**)** | Prospective cohort | Multi-center |  | UK | Probable AIE | 48/48 (children <18 years)  Screened: 111; Enrolled: 48 |
| **de Bruijn 2018 (**[**12**](#_ENREF_12)**)** | Retrospective cohort | Multi-center |  | Netherlands | NMDAR-AIE | 28/28 (children ≤18 years)  Screened: 30; Enrolled: 28 |
| **Hayden 2021 (**[**13**](#_ENREF_13)**)** | Retrospective cohort | Multi-center |  | Hungary | AIE | 8/30 (26.7%; children <18 years; mixed population)  Screened: NR; Enrolled: 8 |
| **Erickson 2020 (**[**14**](#_ENREF_14)**)** | Retrospective cohort | Single-center | North America | US | Encephalitis | 231/231 (children 90 days–18 years)  Screened: 409; Enrolled: 231 |
| **Hariharan 2021 (**[**15**](#_ENREF_15)**)** | Retrospective cohort | Single-center |  | US | AIE | 53/53 (children <18 years)  Screened: NR; Enrolled: 53 |
| **Kang 2023 (**[**16**](#_ENREF_16)**)** | Retrospective cohort | Single-center | Asia Pacific | China | Suspected antibody-mediated CNS autoimmune diseases | 173/173 (children; age in inclusion criteria NR)  Screened: 236; Enrolled: 173 |
| **Pillai 2015 (**[**17**](#_ENREF_17)**)** | Retrospective cohort | Single-center |  | Australia | Encephalitis | 164/164 (children; age in inclusion criteria NR)  Screened: NR; Enrolled: 164 |
| **Woo 2023 (**[**18**](#_ENREF_18)**)** | Retrospective cohort | Single-center |  | Republic of Korea | AIE | 110/110 (children; age in inclusion criteria NR)  Screened: NR; Enrolled: 110 |
| **Kang 2022 (**[**19**](#_ENREF_19)**)** | Retrospective cohort | Single-center |  | China | AIE | 103/103 (children; age in inclusion criteria NR)  Screened: 106; Enrolled: 103 |
| **Deng 2022 (**[**20**](#_ENREF_20)**)** | Retrospective cohort | Multi-center |  | China | AIE | 88/263 (33.5%; children ≤18 years; mixed population)  Screened: NR; Enrolled: 88 |

**Abbreviations:** AIE, autoimmune encephalitis; NMDAR, N-methyl D-aspartate receptor; NR, not reported; UK, United Kingdom; US, United States.

Supplementary Table 9. Studies reporting on proportion data out of NMDAR-AIE used in incidence calculations of post-infection AIE.

| **NMDAR- AIE** | **Numerator** | **Denominator** | **No.^#^** | **Proportion (%)** | **Source** |
| --- | --- | --- | --- | --- | --- |
| **Netherlands** | | | | | |
| **Proportion** | Post-infection NMDAR-AIE | All Dutch children with a confirmed diagnosis of NMDAR-AIE | 28 | 10.71 | de Bruijn 2018 ([12](#_ENREF_12)) |
|  | Post-infection NMDAR-AIE |  | 19 | 31.58 | de Bruijn 2020 ([6](#_ENREF_6)) |

| **Hong Kong** | | | | | |
| --- | --- | --- | --- | --- | --- |
| **Proportion** | Post-infection NMDAR-AIE | Children aged <18 years old who tested positive for anti-NMDAR antibody | 15 | 6.67 | Ho 2018 ([21](#_ENREF_21)) |
| **Republic of Korea** | | | | | |
| **Proportion** | Post-infection NMDAR-AIE | Anti-NMDAR positive AIE | 24 | 4.16 | Woo 2023 ([18](#_ENREF_18)) |

**Abbreviations:** AIE, autoimmune encephalitis; NMDAR, N-methyl D-aspartate receptor. **Footnotes:** #Number of patients in the given denominator.

Supplementary Table 10. Summary of proportion data reported that not used in incidence estimate calculations.

| **Population (numerator)** | **No.^~^** | **Denominator** | **No.^#^** | **Proportion (%)** | **Source** | **Country** |
| --- | --- | --- | --- | --- | --- | --- |
| AIE | 13 | **Acute encephalitis**  **/Encephalitis** | 121 | **10.74** | de Blauw 2020 ([10](#_ENREF_10)) | Netherlands |
| AIE | 657 |  | 6,463 | **10.17** | Parpia 2016 ([22](#_ENREF_22)) | Canada |
| AIE | 7 |  | 95 | **7.37** | Meghan 2020 ([23](#_ENREF_23)) | US |
| NMDAR-AIE | 7 |  | 190 | **3.68** | Dubray 2013 ([24](#_ENREF_24)) | US |
| Immune-mediated encephalitis | 7 |  | 89 | **7.87** | Fowler 2020 ([25](#_ENREF_25)) | Sweden |
| NMDAR-AIE | 2 |  | 40 | **5.00** | Sevilla-Acosta 2020 ([26](#_ENREF_26)) | Costa Rica |
| Presumed AIE (between September 2016–June 2017) | 9 |  | 52 | **17.31** | Galardi 2022 ([27](#_ENREF_27)) | Myanmar |
| Presumed AIE (between December 2017 and August 2018) | 8 |  | 31 | **25.81** |  |  |
| NMDAR-AIE | 1 |  | 41 | **2.44** | Chang 2022 ([28](#_ENREF_28)) | Sri Lanka |
| NMDAR-AIE | 6 |  | 664 | **0.90** | Pommier 2022 ([29](#_ENREF_29)) | Cambodia, Vietnam, Laos, Myanmar |
| Immune-mediated encephalitis | 73 |  | 287 | **25.44** | Britton 2020 ([30](#_ENREF_30)) | Australia |
| Relapsed-AIE | 9 | **Antibody positive AIE** | 19 | **47.37** | Hariharan 2021 ([15](#_ENREF_15)) | US |
| Relapsed NMDAR-AIE | 3 |  | 56 | **5.36** | Pillai 2015 ([17](#_ENREF_17)) | Australia |
| Relapsed Dopamine D2R-AIE | 2 |  | 56 | **3.57** |  |  |
| Relapsed NMDAR-AIE | 3 | **NMDAR-AIE** | 28 | **10.71** | De Bruijn 2018 ([12](#_ENREF_12)) | Netherlands |
|  | 7 |  | 31 | **22.58** | Wright 2015 ([31](#_ENREF_31)) | UK |
|  | 2 |  | 16 | **12.50** | Jones 2017 ([7](#_ENREF_7)) | New Zealand |
| Antibody-positive LE | 5 | **Idiopathic LE** | 10 | **50.00** | Chou 2013 ([32](#_ENREF_32)) | Taiwan |
| Antibody-negative LE | 5 |  |  | **50.00** |  |  |
| Anti-amphiphysin positive LE | 3 |  |  | **30.00** |  |  |
| Anti-GAD positive LE | 3 |  |  | **30.00** |  |  |
| Anti-amphiphysin and anti-GAD positive LE | 1 |  |  | **10.00** |  |  |
| NMDAR-AIE | 4 | **All PICU admissions** | 178,686 | **0.002** | Iro 2019 ([33](#_ENREF_33)) | England and Wales |
| Rasmussen's encephalitis | 2 |  |  | **0.001** |  |  |
| LE | 1 |  |  | **0.001** |  |  |
| Hashimoto's encephalitis | 1 |  |  | **0.001** |  |  |
| AIE (Unspecified) | 1 |  |  | **0.001** |  |  |
| Anti-NMDAR immune mediated encephalitis | 3 | **Immune-mediated encephalitis** | 7 | **42.86** | Fowler 2020 ([25](#_ENREF_25)) | Sweden |
| Post-infection anti-NMDAR immune mediated encephalitis | 2 |  |  | **28.57** |  |  |
| Anti-NMDAR immune mediated encephalitis + VZV infection | 1 |  |  | **14.29** |  |  |
| Hashimoto's encephalitis | 1 |  |  | **14.29** |  |  |
| NMDAR-AIE | 17 |  | 73 | **23.29** | Britton 2020 ([30](#_ENREF_30)) | Australia |
| GAD-AIE | 1 |  |  | **1.37** |  |  |
| NMDAR-AIE | 1 |  | 15 | **6.67** | Uslu 2019 ([34](#_ENREF_34)) | Turkey |
| CASPR2-AIE | 1 |  |  | **6.67** |  |  |
| GAD-AIE | 8 |  |  | **53.33** |  |  |
| TPO-AIE | 5 |  |  | **33.33** |  |  |
| NMDAR-AIE | 4 |  | 83 | **4.82** | Iro 2019 ([33](#_ENREF_33)) | England and Wales |
| Rasmussen's encephalitis | 2 |  |  | **2.41** |  |  |
| Limbic encephalitis | 1 |  |  | **1.20** |  |  |
| Hashimoto's encephalitis | 1 |  |  | **1.20** |  |  |
| AIE (Unspecified) | 1 |  |  | **1.20** |  |  |
| Antibody positive AIE | 18 | **Probable/Suspected AIE** | 375 | **4.80** | Boesen 2019 ([4](#_ENREF_4)) | Denmark |
| AIE (serum samples) | 251 |  | 5,649 | **4.44** | Kunchok 2022 ([35](#_ENREF_35)) | US |
| AIE (CSF samples) | 282 |  | 5,136 | **5.49** |  |  |
| NMDAR-AIE | 8 |  | 9 | **88.89** | Musso 2018 ([36](#_ENREF_36)) | Italy |
| NMDAR-AIE (tested using Euroimmune AIE panel) | 5 |  | 8 | **62.50** | Galardi 2022 ([27](#_ENREF_27)) | Myanmar |
| NMDAR-AIE (confirmation samples sent to Barcelona) | 4 |  | 8 | **50.00** |  |  |
| NMDAR-AIE | 7 |  | 20 | **35.00** | Lizcano-Meneses 2021 ([37](#_ENREF_37)) | Brazil |
| GAD-AIE | 1 |  | 20 | **5.00** |  |  |
| Antibody positive AIE | 19 | **AIE (clinical diagnosis)** | 53 | **35.85** | Hariharan 2021 ([15](#_ENREF_15)) | US |
| NMDAR-AIE | 97 | **Antibody-mediated CNS autoimmune diseases** | 173 | **56.07** | Kang 2023 ([16](#_ENREF_16)) | China |
| CASPR-AIE | 5 |  |  | **2.89** |  |  |
| GABABR-AIE | 2 |  |  | **1.15** |  |  |
| LGI1-AIE | 1 |  |  | **0.58** |  |  |
| NMDAR+CASPR2-AIE | 1 |  |  | **0.58** |  |  |
| CASPR2 + GABABR AIE | 1 |  |  | **0.58** |  |  |
| MOG antibody-associated disorders | 48 |  |  | **27.75** |  |  |
| Autoimmune GFAP-astrocytopathy | 30 |  |  | **17.34** |  |  |
| Anti-AQP4 autoantibodies | 3 |  |  | **17.34** |  |  |
| AB-associated IBrainD | 16 | **Pediatric patients with IBrainD** | 169 | **9.47** | Bigi 2015 ([38](#_ENREF_38)) | Canada |
| NMDAR-AIE | 9 | **Pediatric patients with AB-associated IBrainD** | 16 | **56.25** |  |  |
| Hashimoto encephalitis | 2 |  |  | **12.50** |  |  |
| GAD65-AIE | 1 |  |  | **6.25** |  |  |
| Neuronal autoantibody-positive EE | 6 | **Patients with EE** | 50 | **12.00** | Tekturk 2018 ([39](#_ENREF_39)) | Turkey |
| Anti-NMDAR positive EE | 2 |  |  | **4.00** |  |  |
| Anti-GAD positive EE | 1 |  |  | **2.00** |  |  |
| Anti-GABAAR positive EE | 1 |  |  | **2.00** |  |  |
| Anti-CASPR2 positive EE | 1 |  |  | **2.00** |  |  |
| Anti-GlyR positive EE | 1 |  |  | **2.00** |  |  |
| NMDAR-AIE | 89 | **Probable or definite AIE** | 103 | **86.41** | Zhang 2019 ([40](#_ENREF_40)) | China |
| LGI1-AIE | 2 |  |  | **1.94** |  |  |
| CASPR2-AIE | 1 |  |  | **0.97** |  |  |
| Autoantibody-negative but probable AIE | 11 |  |  | **10.68** |  |  |

**Abbreviations:** AIE, autoimmune encephalitis; AB-associated IBrainD, Antibody-associated inflammatory brain diseases; AQP4, Aquaporin 4; CASPR2, Contactin-associated protein-like 2; CSF, cerebrospinal fluid; D2R, Dopamine Receptor 2; GABAR, Gamma-aminobutyric acid receptors; EE, epileptic encephalopathy; GAD, Glutamic acid decarboxylase; GFAP, Glial fibrillary acidic protein; GlyR, Glycine receptor; LE, Limbic encephalitis; LGI1, Leucine-rich glioma inactivated 1; MOG, Myelin oligodendrocyte glycoprotein NMDAR, N-methyl D-aspartate receptor; PICU, pediatric intensive care unit; TPO, Thyroid peroxidase; VZV, Varicella zoster virus. **Footnotes:** ~Number of patients in the given numerator; #Number of patients in the given denominator.

Supplementary Table 11. Calculated incidence data of post-infection NMDAR-AIE.

|  | Netherlands | UK | Hungary | US | China | Hong Kong | Republic of Korea | Australia |
| --- | --- | --- | --- | --- | --- | --- | --- | --- |
| Based on proportion in definite NMDAR cases | | | | | | | | |
| Post-infection NMDAR-AIE | 0.1–1.3* | - | - | - | - | 0.2 | 0.1 | - |

**Abbreviations:** AIE, autoimmune encephalitis; NMDAR, N-methyl D-aspartate receptor; UK. United Kingdom; US, United States. **Footnotes:** *Lower and upper limit incidence estimates calculated using proportion data from different studies.

Supplementary Table 12. Summary of study characteristics for studies reporting proportion data not used in the calculation of incidence estimates.

| **Study name** | **Study design** | **Single-center/ multi-center** | **Continent** | **Countries** | **Primary condition in population** | **No. of children included/total population** |
| --- | --- | --- | --- | --- | --- | --- |
| **Iro 2019 (**[**33**](#_ENREF_33)**)** | Retrospective cohort | Multi-center | Europe | UK | Encephalitis | 1,031/1,031 (children <18 years)  Screened: NR; Enrolled: 1,031 |
| **Fowler 2020 (**[**25**](#_ENREF_25)**)** | Prospective cohort | Single-center |  | Sweden | Acute encephalitis | 89/89 (children 28 days–17 years)  Screened: NR; Enrolled: 89 |
| **Nissen 2022 (**[**41**](#_ENREF_41)**)** | Retrospective cohort | Multi-center |  | Denmark | NMDAR-AIE | 11/55 (20%; mixed population; age in inclusion criteria NR)  Screened: NR; Enrolled: 11 |
| **Musso 2018* (**[**36**](#_ENREF_36)**)** | Retrospective cohort | Single-center |  | Italy | Suspected AIE | NR (mixed population; age in inclusion criteria NR; stated inclusion of children)  NR (Screened: NR; Enrolled: NR) |
| **Dubray 2013 (**[**24**](#_ENREF_24)**)** | Retrospective cohort | Single-center | United States | US | Encephalitis | 190/190 (children; age in inclusion criteria NR)  Screened: 305; Enrolled: 190 |
| **Bigi 2015 (**[**38**](#_ENREF_38)**)** | Retrospective cohort | Single-center |  | Canada | Pediatric Ab-associated IBrainD | 169/169 (children ≤18 years)  Screened: NR; Enrolled: 169 |
| **Meghan 2020 (**[**23**](#_ENREF_23)**)** | Retrospective cohort | Single-center |  | US | Acute encephalitis | 95/95 (children 6 months–18 years)  Screened: 937; Enrolled: 95 |
| **Kunchok 2022* (**[**35**](#_ENREF_35)**)** | Retrospective cohort | Single-center |  | US | Suspected AIE | NR (mixed population; age in inclusion criteria NR; stated inclusion of children)  NR (Screened: NR; Enrolled: NR) |
| **Pommier 2022 (**[**29**](#_ENREF_29)**)** | Prospective cohort | Multi-center | Asia Pacific | Cambodia, Vietnam, Laos, Myanmar | Encephalitis | 664/664 (children 28 days–16 years)  Screened: 4,878; Enrolled: 664 |
| **Britton 2020 (**[**30**](#_ENREF_30)**)** | Prospective cohort | Multi-center |  | Australia | Suspected encephalitis | 526/526 (children ≤14 years)  Screened: NR; Enrolled: 526 |
| **Zhang 2019 (**[**40**](#_ENREF_40)**)** | Retrospective cohort | Multi-center |  | China | AIE | 103/103 (children ≤18 years)  Screened: NR; Enrolled: 103 |
| **Galardi 2022 (**[**27**](#_ENREF_27)**)** | Prospective cohort | Single-center |  | Myanmar | Encephalitis including presumed infectious encephalitis and presumed AIE | 103/103 (children aged ≤12 years)  Screened: NR; Enrolled: 103 |
| **Suleiman 2011 (**[**42**](#_ENREF_42)**)** | Retrospective cohort | Single-center |  | Australia | Unexplained encephalitis presenting with status epilepticus and refractory seizures | 10/10 (children; age in inclusion criteria NR)  Screened: NR; Enrolled: 10 |
| **Chou 2013 (**[**32**](#_ENREF_32)**)** | Retrospective case control/matched | Single-center |  | Taiwan | Idiopathic LE | 10/10 (children <18 years)  Screened: NR; Enrolled: 10 |
| **Shan 2021* (**[**43**](#_ENREF_43)**)** | Retrospective cohort | Multi-center |  | China | AIE | NR (mixed population; age in inclusion criteria NR; stated inclusion of children)  Screened: NR; Enrolled: 778 |
| **Chang 2022 (**[**28**](#_ENREF_28)**)** | Prospective cohort | Multi-center |  | Sri Lanka | Encephalitis/meningoencephalitis | 41/99 (41.4%; children ≤12 years; mixed population)  Screened: NR; Enrolled: 41 |
| **Reyes 2021 (**[**44**](#_ENREF_44)**)** | Retrospective cohort | Single-center |  | Philippines | Definite AIE | 6/18 (33.3%; mixed population; age in inclusion criteria NR; stated inclusion of children)  Screened: NR; Enrolled: 6 |
| **Uslu 2019 (**[**34**](#_ENREF_34)**)** | Retrospective cohort | Single-center | Middle East | Turkey | AIE | 15/15 (children; age in inclusion criteria NR)  Screened: NR; Enrolled: 15 |
| **Tekturk 2018 (**[**39**](#_ENREF_39)**)** | Retrospective cohort | Single-center |  | Turkey | EE | 6/50 (12%; mixed population; age in inclusion criteria NR; stated inclusion of children)  Screened: NR; Enrolled: 6 |
| **Sevilla-Acosta 2020 (**[**26**](#_ENREF_26)**)** | Prospective cohort | Single-center | South America | Costa Rica | Acute encephalitis | 40/40 (children 1 month–13 years)  Screened: 46; Enrolled: 40 |
| **Lizcano-Meneses 2021 (**[**37**](#_ENREF_37)**)** | Retrospective cohort | Multi-center |  | Brazil | Probable AIE | NR (children <18 years; mixed population)  Screened: NR; Enrolled: 101 |

**Abbreviations:** AIE, autoimmune encephalitis; EE, epileptic encephalopathy; LE: limbic encephalitis; NMDAR, N-methyl D-aspartate receptor; NR, not reported; UK, United Kingdom; US, United States.

**Supplementary Figure 1:** Summary of the quality assessments conducted using the JBI Critical Appraisal Checklist for studies reporting directly incidence and proportion data used in the calculations.


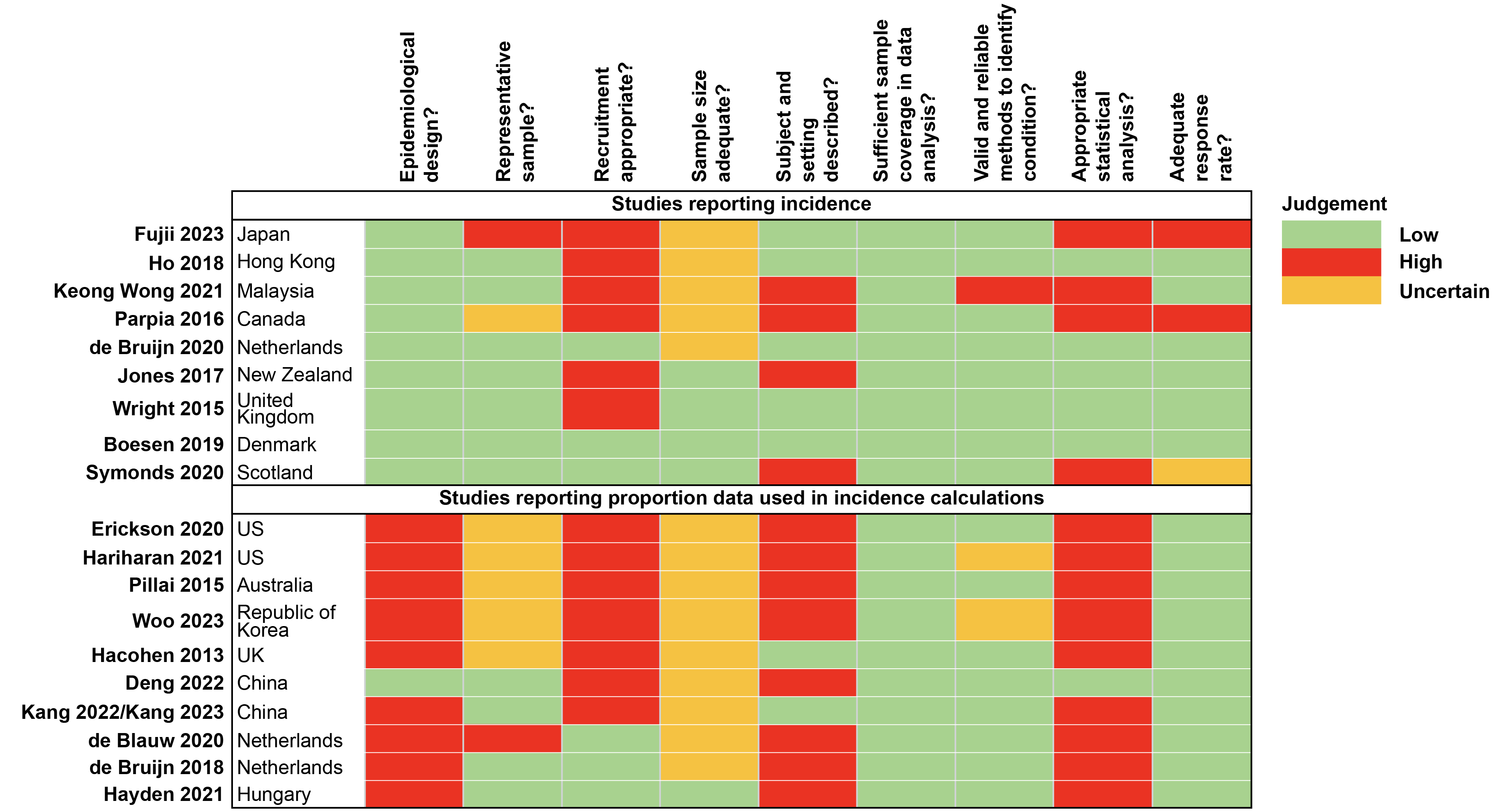


**Abbreviations**: JBI, Joanna Briggs Institute; UK, United Kingdom; US, United States.

Supplementary Figure 2. Summary of the quality assessments conducted using the JBI Critical Appraisal Checklist for studies reporting proportion data not used in the calculations.


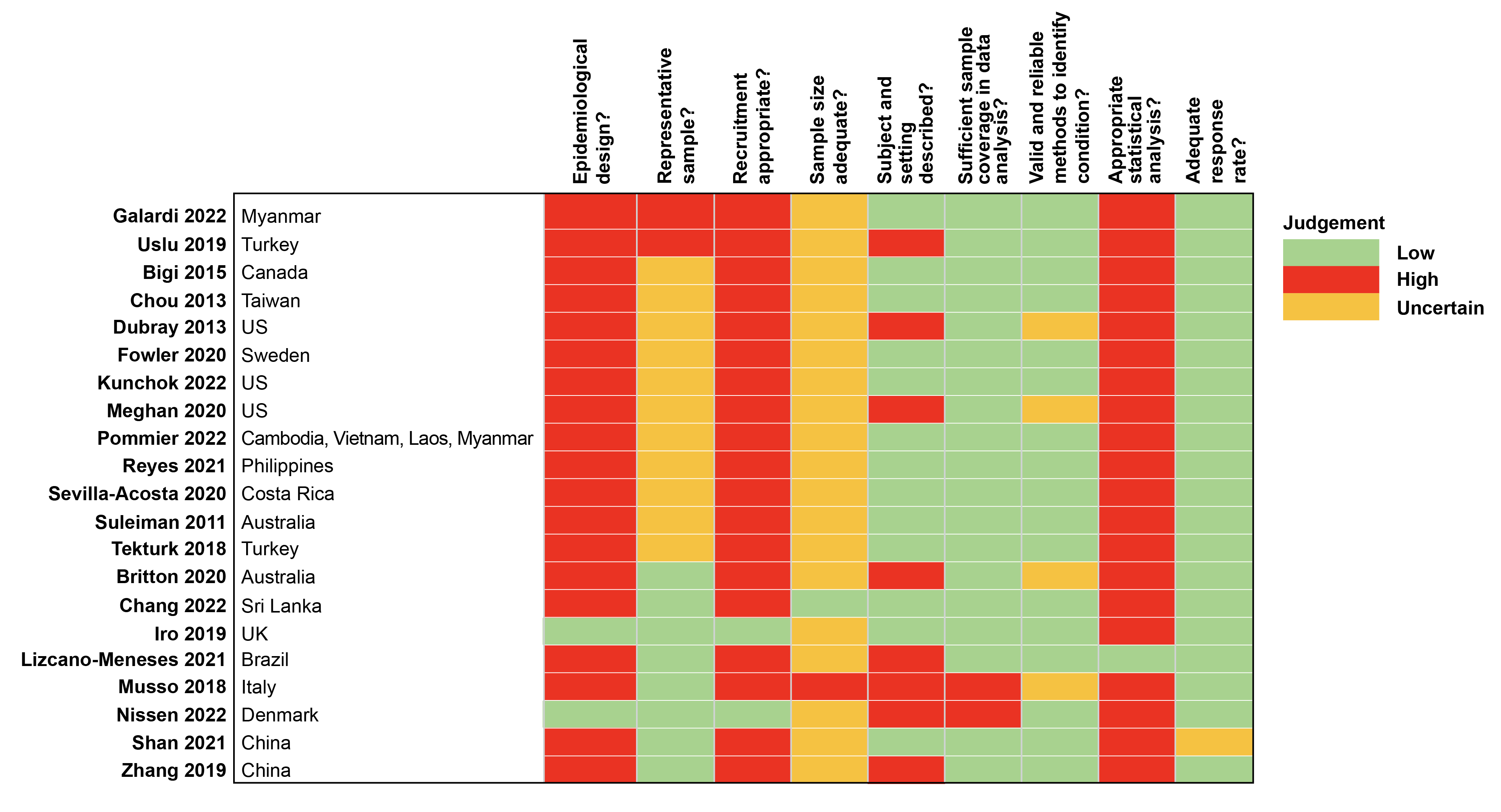


**Abbreviations**: JBI, Joanna Briggs Institute; UK, United Kingdom; US, United States**.**

**References:**

1. Tenny S BS. Incidence. [Updated 2023 Apr 10]. StatPearls [Internet]. Treasure Island (FL): StatPearls 2023 [Available from: <https://www.ncbi.nlm.nih.gov/books/NBK430746/>.

2. Denmark S. Population: 1st January 2018 [Available from: <https://extranet.dst.dk/pyramide/pyramide.htm#!y=2018&a=18,66&v=2&o=2023&l=en&g>.

3. 2022 SsC. Scotland's Census 2022- Rounded Population Estimates 2022 [Available from: <https://www.scotlandscensus.gov.uk/2022-results/scotland-s-census-2022-rounded-population-estimates/>.

4. Boesen MS, Born AP, Lydolph MC, Blaabjerg M, Borresen ML. Pediatric Autoimmune Encephalitis in Denmark During 2011-17: A Nationwide Multicenter Population-Based Cohort Study. Eur J Paediatr Neurol. 2019;23(4):639-652.

5. Symonds JD, Moloney TC, Lang B, McLellan A, O'Regan ME, Macleod S, et al. Neuronal Antibody Prevalence in Children With Seizures Under 3 Years: A Prospective National Cohort. Neurology. 2020;95(11):E1590-E1598.

6. de Bruijn M, Bruijstens AL, Bastiaansen AEM, van Sonderen A, Schreurs MWJ, Sillevis Smitt PAE, et al. Pediatric Autoimmune Encephalitis: Recognition and Diagnosis. Neurology Neuroimmunology & Neuroinflammation. 2020;7(3):05.

7. Jones HF, Mohammad SS, Reed PW, Dunn PPJ, Steele RH, Dale RC, et al. Anti-N-Methyl-D-Aspartate Receptor Encephalitis in Maori and Pacific Island Children in New Zealand. Developmental Medicine & Child Neurology. 2017;59(7):719-724.

8. Keong Wong C, Pillai P, Hor J, Loo Y, Hock S, Shirley L, et al. High Incidence of NMDAR Encephalitis Among Austronesians: A Population-Based Study in Sabah, Malaysia. Eur J Neurol. 2021;28(SUPPL 1):183.

9. Fujii K, Shiohama T, Uchida T, Ikehara H, Fukuhara T, Sawada D, et al. Nationwide survey of childhood Guillain-Barre syndrome, Fisher syndrome, and Bickerstaff Brainstem Encephalitis in Japan. Brain & Development. 2023;45(1):16-25.

10. de Blauw D, Bruning AHL, Busch CBE, Kolodziej LM, Jansen NJG, van Woensel JBM, et al. Epidemiology and Etiology of Severe Childhood Encephalitis in The Netherlands. Pediatr Infect Dis J. 2020;39(4):267–272.

11. Hacohen Y, Wright S, Waters P, Agrawal S, Carr L, Cross H, et al. Paediatric Autoimmune Encephalopathies: Clinical Features, Laboratory Investigations and Outcomes in Patients With or Without Antibodies to Known Central Nervous System Autoantigens. Journal of Neurology, Neurosurgery & Psychiatry. 2013;84(7):748-755.

12. de Bruijn M, Aarsen FK, van Oosterhout MP, van der Knoop MM, Catsman-Berrevoets CE, Schreurs MWJ, et al. Long-Term Neuropsychological Outcome Following Pediatric Anti-NMDAR Encephalitis. Neurology. 2018;90(22):e1997-e2005.

13. Hayden Z, Bone B, Orsi G, Szots M, Nagy F, Csepany T, et al. Clinical Characteristics and Outcome of Neuronal Surface Antibody-Mediated Autoimmune Encephalitis Patients in a National Cohort. Frontiers in Neurology. 2021;12:611597.

14. Erickson TA, Muscal E, Munoz FM, Lotze T, Hasbun R, Brown E, et al. Infectious and Autoimmune Causes of Encephalitis in Children. Pediatrics. 2020;145(6):06.

15. Hariharan P, Crespo D, Rathore G. Seropositive Pediatric Autoimmune Encephalitis: A Single Center Experience. Neurology. Conference: 73rd Annual Meeting of the American Academy of Neurology, AAN. 2021;96(15 SUPPL 1).

16. Kang Q, Liao H, Yang L, Fang H, Ning Z, Liao C, et al. Clinical Analysis of 173 Pediatric Patients With Antibody-Mediated Autoimmune Diseases of the Central Nervous System: A Single-Center Cohort Study. Frontiers in Immunology. 2023;14:1140872.

17. Pillai SC, Hacohen Y, Tantsis E, Prelog K, Merheb V, Kesson A, et al. Infectious and Autoantibody-Associated Encephalitis: Clinical Features and Long-Term Outcome. Pediatrics. 2015;135(4):e974-984.

18. Woo H, Shim Y, Chae JH, Kim KJ, Lim BC. Seizure Evolution and Outcome in Pediatric Autoimmune Encephalitis. Pediatric Neurol. 2023;139:35-42.

19. Kang Q, Liao H, Yang L, Fang H, Hu W, Wu L. Clinical Characteristics and Short-Term Prognosis of Children With Antibody-Mediated Autoimmune Encephalitis: A Single-Center Cohort Study. Frontiers in Pediatrics. 2022;10 880693.

20. Deng Q, Liu Y, Mao Z, Chen Y, Ping Y, Zhu G, et al. The Antibody Assay in Suspected Autoimmune Encephalitis From Positive Rate to Test Strategies. Front Immunol. 2022;13:803854.

21. Ho ACC, Chan SHS, Chan E, Wong SSN, Fung STH, Cherk SWW, et al. Anti-N-Methyl-D-Aspartate Receptor Encephalitis in Children: Incidence and Experience in Hong Kong. Brain Dev. 2018;40(6):473-479.

22. Parpia AS, Li Y, Chen C, Dhar B, Crowcroft NS. Encephalitis, Ontario, Canada, 2002-2013. Emerging Infectious Diseases. 2016;22(3):426-432.

23. Meghan M, Kavita T. Acute Encephalitis in the Pediatric Population of Western Pennsylvania. Child Neurology Society. 2020:56.

24. Dubray K, Anglemyer A, Labeaud AD, Flori H, Bloch K, Joaquin KS, et al. Epidemiology, Outcomes and Predictors of Recovery in Childhood Encephalitis: A Hospital-Based Study. Pediatr Infect Dis J. 2013;32(8):839-844.

25. Fowler A, Ygberg S, Svensson E, Bergman K, Cooray G, Wickstrom R. Prospective Evaluation of Childhood Encephalitis: Predisposing Factors, Prevention and Outcome. Pediatr Infect Dis J. 2020;39(12):e417-e422.

26. Sevilla-Acosta F, Gutierrez-Mata A, Yock-Corrales A, Bogantes-Ledezma S, Perez-Corrales C, Camacho-Badilla K. Epidemiology, Etiology and Clinical Aspects of Childhood Acute Encephalitis in a Tertiary Pediatric Hospital in Costa Rica. Pediatr Infect Dis J. 2020;40(3):186-190.

27. Galardi MM, Sowa GM, Crockett CD, Robert R, Smith AE, Shwe EE, et al. Pathogen and Antibody Identification in Children with Encephalitis in Myanmar. Ann Neurol. 2022;28.

28. Chang T, Moloney T, Jacobson L, Malavige N, Lohitharajah J, Wanigasinghe J, et al. Significance of Neuronal Autoantibodies in Comparison to Infectious Etiologies Among Patients Presenting with Encephalitis in a Region with a High Prevalence of Infections. Ann Indian Acad Neurol. 2022;25(3):473-478.

29. Pommier JD, Gorman C, Crabol Y, Bleakley K, Sothy H, Santy K, et al. Childhood Encephalitis in the Greater Mekong Region (the SouthEast Asia Encephalitis Project): A Multicentre Prospective Study. The Lancet Global Health. 2022;10(7):e989-e1002.

30. Britton PN, Dale RC, Blyth CC, Clark JE, Crawford N, Marshall H, et al. Causes and Clinical Features of Childhood Encephalitis: A Multicenter, Prospective Cohort Study. Clin Infect Dis. 2020;70(12):2517-2526.

31. Wright S, Hacohen Y, Jacobson L, Agrawal S, Gupta R, Philip S, et al. N-Methyl-D-Aspartate Receptor Antibody-Mediated Neurological Disease: Results of a UK-Based Surveillance Study in Children. Archives of Disease in Childhood. 2015;100(6):521-526.

32. Chou IJ, Wang HS, Lin JJ, Kuo CF, Lin KL, Chou ML, et al. Limbic Encephalitis in Taiwanese Children and Adolescence: A Single Center Study. Pediatrics & Neonatology. 2013;54(4):246-253.

33. Iro MA, Sadarangani M, Nickless A, Kelly DF, Pollard AJ. A Population-based Observational Study of Childhood Encephalitis in Children Admitted to Pediatric Intensive Care Units in England and Wales. Pediatr Infect Dis J. 2019;38(7):673-677.

34. Uslu I, Varol F, Guler S, Cam H, Saltik S. Electroclinical Features and Seizure Outcome of Peadiatric Patients With Immune Mediated Encephalopathy. Archives of Disease in Childhood. 2019;104(Supplement 3):A349-A350.

35. Kunchok A, McKeon A, Zekeridou A, Flanagan EP, Dubey D, Lennon VA, et al. Autoimmune/Paraneoplastic Encephalitis Antibody Biomarkers: Frequency, Age, and Sex Associations. Mayo Clinic Proceedings. 2022;97(3):547-559.

36. Musso G, Seguso M, Gallo N, Plebani M. Laboratory Phenotypes of Antibodymediated Encephalitis: A Retrospective Analysis in Secondary and Tertiary Care University-Hospital Laboratory. Biochimica Clinica. 2018;42(Supplement 1):S137.

37. Lizcano-Meneses A, Watanabe N, von Glehn F, Barbosa R, de Albuquerque M, Yassuda C, et al. Clinical Variables That Help in Predicting the Presence of Autoantibodies in Patients With Acute Encephalitis. Seizure. 2021;90:117-122.

38. Bigi S, Hladio, M., Twilt, M., Dalmau, J., & Benseler, S. M. The Growing Spectrum of Antibody-Associated Inflammatory Brain Diseases in Children. Neurology Neuroimmunology & Neuroinflammation. 2015;2(3):e29.

39. Tekturk P, Baykan B, Erdag E, Peach S, Sezgin M, Yapici Z, et al. Investigation of Neuronal Auto-Antibodies in Children Diagnosed With Epileptic Encephalopathy of Unknown Cause. Brain Dev. 2018;40(10):909-917.

40. Zhang W, Wang X, Shao N, Ma R, Meng H. Seizure Characteristics, Treatment, and Outcome in Autoimmune Synaptic Encephalitis: A Long-Term Study. Epilepsy Behav. 2019;94:198-203.

41. Nissen MS, Orvik MS, Nilsson AC, Ryding M, Lydolph M, Blaabjerg M. NMDA-Receptor Encephalitis in Denmark From 2009 to 2019: A National Cohort Study. J Neurol. 2022;269(3):1618-1630.

42. Suleiman J, Brenner T, Gill D, Brilot F, Antony J, Vincent A, et al. VGKC Antibodies in Pediatric Encephalitis Presenting With Status Epilepticus. Neurology. 2011;76(14):1252-1255.

43. Shan W, Yang H, Wang Q. Neuronal Surface Antibody-Medicated Autoimmune Encephalitis (Limbic Encephalitis) in China: A Multiple-Center, Retrospective Study. Front Immunol. 2021;12:621599.

44. Reyes NGD, Espiritu AI, Agabao JF, Abejero JEE, Salonga-Quimpo RAM, Cabral-Lim LI, et al. Autoimmune Encephalitis in a Tertiary Hospital in the Philippines. J Clin Neurosci. 2021;90:191-198.
